# Single-cell synaptome mapping: its technical basis and applications in critical period plasticity research

**DOI:** 10.3389/fncir.2024.1523614

**Published:** 2024-12-11

**Authors:** Motokazu Uchigashima, Takayasu Mikuni

**Affiliations:** ^1^Department of Cellular Neuropathology, Brain Research Institute, Niigata University, Niigata, Japan; ^2^International Research Center for Neurointelligence (WPI-IRCN), The University of Tokyo, Tokyo, Japan

**Keywords:** endogenous proteins, synapse, synaptome, single cell, critical period plasticity, intrabody, CRISPR/Cas9, genome editing

## Abstract

Our brain adapts to the environment by optimizing its function through experience-dependent cortical plasticity. This plasticity is transiently enhanced during a developmental stage, known as the “critical period,” and subsequently maintained at lower levels throughout adulthood. Thus, understanding the mechanism underlying critical period plasticity is crucial for improving brain adaptability across the lifespan. Critical period plasticity relies on activity-dependent circuit remodeling through anatomical and functional changes at individual synapses. However, it remains challenging to identify the molecular signatures of synapses responsible for critical period plasticity and to understand how these plasticity-related synapses are spatiotemporally organized within a neuron. Recent advances in genetic tools and genome editing methodologies have enabled single-cell endogenous protein labeling in the brain, allowing for comprehensive molecular profiling of individual synapses within a neuron, namely “single-cell synaptome mapping.” This promising approach can facilitate insights into the spatiotemporal organization of synapses that are sparse yet functionally important within single neurons. In this review, we introduce the basics of single-cell synaptome mapping and discuss its methodologies and applications to investigate the synaptic and cellular mechanisms underlying circuit remodeling during the critical period.

## 1 Introduction

The brain adapts to the surrounding environment by optimizing its functions in an experience-dependent manner. The neural plasticity that underlies this adaptation peaks at a narrow developmental time window, known as the “critical period” (Hubel and Wiesel, 1970). The brain subsequently reduces its adaptability, maintaining it at lower levels across adulthood (Hübener and Bonhoeffer, 2014). This developmental change enables efficient acquisition of new skills, such as learning a second language in childhood, but also limits recovery from ischemic or traumatic brain damages in adulthood (Bjorklund, 2022). Thus, understanding the mechanisms that govern the opening, maintenance, and closure of the developmental critical period, as well as how the critical period reopens in adulthood, is crucial for advancing our knowledge of brain plasticity and its applications in neurological recovery (Hensch, 2004; Bavelier et al., 2010).

The cellular mechanism underlying critical period plasticity involves activity-dependent circuit remodeling through anatomical and functional changes at individual synapses. The visual cortex, extensively studied as a model of critical period plasticity (Hensch, 2005; Espinosa and Stryker, 2012), requires both Hebbian and non-Hebbian synaptic plasticity for circuit remodeling (Mrsic-Flogel et al., 2007; Yoon et al., 2009; Ranson et al., 2012). Structural changes of dendritic spines, which are protrusions that form excitatory synapses, are enhanced in pyramidal cells during early life (Majewska and Sur, 2003). These synaptic phenotypes vary across individual synapses (Majewska and Sur, 2003; Sun et al., 2019). However, previous studies have often relied on averaged data from a limited number of synapses, potentially overlooking specific synaptic subpopulations that contribute to circuit remodeling. Therefore, comprehensive profiling of individual synapses is essential for a better understanding of the circuit remodeling in critical period plasticity. In this review, we introduce a powerful approach for molecular profiling of individual synapses within a neuron, called “single-cell synaptome mapping.” We also discuss the technical methodologies and applications of this approach to investigate the synaptic and cellular mechanisms underlying circuit remodeling during the critical period in the mouse brain.

## 2 Single-cell synaptome: comprehensive molecular profiles of individual synapses within a neuron

The term “synaptome” is derived from “synapse” and “ome” (meaning “totality”). It was originally used as a part of the connectome, a comprehensive map of neuronal connections between different neurons or regions within the central nervous system (DeFelipe, 2010). Recently, the concept of the synaptome has expanded to include the synapse proteome, comprising over 1,000 protein species essential for synaptic functions (Koopmans et al., 2019; van Oostrum et al., 2023).

**Figure 1:**
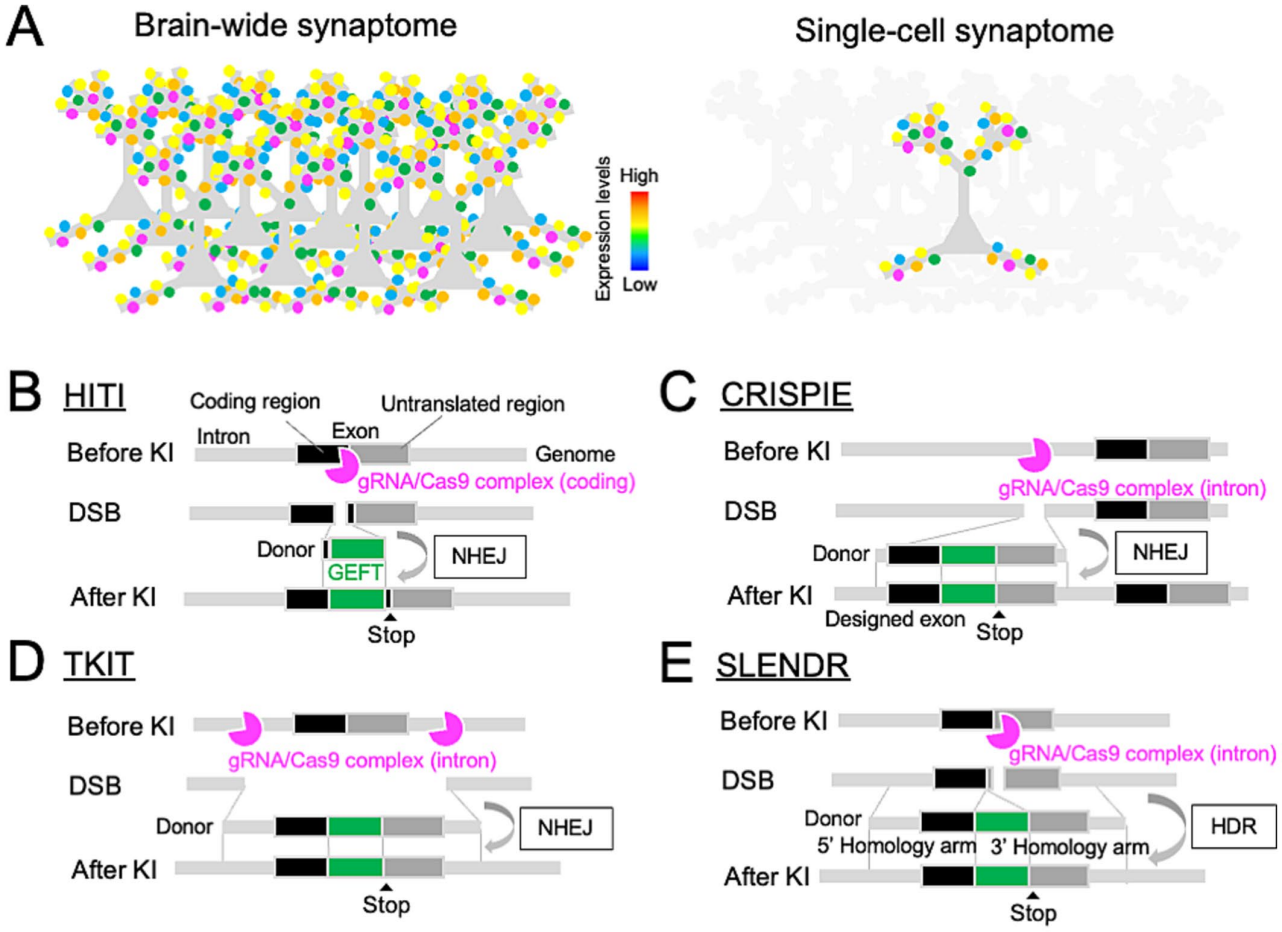
Genome editing toolbox for single-cell endogenous protein labeling in the brain. **(A)** Comparison of brain-wide (top) and single-cell (bottom) synaptome mapping based on endogenous protein labeling at individual synapses. **(B–E)** Design of genome editing-mediated GEFT KI through the NHEJ-mediated HITI **(B)**, CRISPIE **(C)**, or TKIT **(D)**, and HDR-mediated SLENDR **(E)**, allowing for single-cell endogenous protein labeling with GEFTs in the brain.

Spatial mapping of endogenous proteins at individual synapses is crucial for understanding synaptic protein-based synaptome. Immunohistochemistry is the most common method for labeling endogenous proteins in brain tissues. However, non-specific immunoreactivity and limited antibody penetration often cause challenges to quantitative accuracy (Watanabe et al., 1998), despite recent advances such as computational antibody design (Kim et al., 2023), glyoxal fixation (Konno et al., 2023), and chemical and physical modifications of antibodies and fixed tissues (Yau et al., 2023). Grant and colleagues developed a methodological pipeline for analyzing the molecular profiles of individual synapses across the brain, referred to as the brain-wide synaptome (Figure 1A) (Zhu et al., 2018; Cizeron et al., 2020; Bulovaite et al., 2022; Tomas-Roca et al., 2022; Koukaroudi et al., 2024). This is based on large-scale, high-resolution, quantitative measurements of the expression levels of major endogenous postsynaptic proteins (e.g., postsynaptic scaffolds PSD95 and SAP102) genetically labeled with fluorescent tags across the mouse brain. Mathematical analyses revealed synaptome maps that vary depending on cell types, brain regions, ages, and disease states. However, understanding the intricate synaptome at the cellular level remains a challenge due to the remarkable heterogeneity of neurons within the brain.

Single-cell synaptome mapping offers a promising approach for dissecting the complexity of the brain-wide synaptome at cellular levels. In the neocortex, single pyramidal cells receive inputs from other neurons via thousands of synapses on dendritic branches (Iascone et al., 2020). Comprehensive molecular profiling of these synapses within individual neurons, which is referred to as “single-cell synaptome,” enables researchers to explore the molecular diversity of synapses at single-cell levels in the brain (Figure 1A). This approach can help identify synapses that are few in number but functionally impactful within a neuron, and reveal the spatiotemporal organization of individual synaptic profiles that underlies the single-neuron computation. Therefore, single-cell synaptome mapping can gain a deeper insight into the synaptic and cellular mechanisms underlying circuit remodeling during the critical period.

## 3 Technical methodologies of single-cell synaptome mapping

Single-cell synaptome mapping requires single-cell endogenous protein labeling, volumetric fluorescence imaging, and quantitative signal detection in living or fixed mouse brains. Genetically-encoded fluorescent tags (GEFTs) with brightness and photostability are suitable for these requirements. Indeed, the brain-wide synaptome analysis described by Grant and colleagues is based on genetic labeling of endogenous proteins with GEFTs, such as mEGFP or HaloTag (Zhu et al., 2018; Cizeron et al., 2020; Bulovaite et al., 2022; Tomas-Roca et al., 2022; Koukaroudi et al., 2024). Recent technological advances in genetic tools and genome editing methodologies have revolutionized single-cell endogenous protein labeling. In this section, we provide an overview of several methods for single-cell endogenous labeling with GEFTs in the mammalian brain.

### 3.1 Intrabody-mediated endogenous protein labeling

Intrabodies are small recombinant antibody-like proteins genetically encoded for the heterologous expression, allowing for efficient access to intact endogenous target proteins within a living neuron transfected with intrabodies (Trimmer, 2022). Different types of intrabodies are available, such as single-chain variable fragments (scFvs), nanobodies (nAbs), and recombinant fibronectin-based molecules (monobodies or FingR). Unlike normal full-length antibodies (IgG) with two pairs of heavy and light chains which contain multiple variable and constant domains, a scFv consists of a single pair of heavy- and light-chain variable domains which tether together via a peptide linker to make a binding site to antigen epitopes (Bird et al., 1988; Huston et al., 1988; Fukata et al., 2013; Kabayama et al., 2020). nAbs are based on the variable domain of camel single-chain antibodies, which are sufficient for binding to antigen epitopes (Hamers-Casterman et al., 1993; Dong et al., 2019; Kilisch et al., 2023; Queiroz Zetune Villa Real et al., 2023). Monobodies or FingR are genetically engineered from human 10th fibronectin type III domains to bind to target proteins via mRNA display (Koide et al., 1998; Karatan et al., 2004; Gross et al., 2013; Mora et al., 2013). For single-cell endogenous protein labeling, these intrabodies are fused with GEFTs, and genetically introduced into specific cells. Since this approach relies on the overexpression of intrabodies, target-binding intrabodies may be outnumbered by unbound intrabodies, which can reduce the signal-to-noise ratio. Transcriptional feedback control can help limit excess unbound intrabodies within the cell (Gross et al., 2013).

Many intrabodies have been developed to target various synaptic proteins, such as CaMKIIα (Mora et al., 2013), PSD95 (Fukata et al., 2013; Gross et al., 2013; Rimbault et al., 2024), Gephyrin (Gross et al., 2013; Dong et al., 2019), and Synaptotagmin-1 (Kabayama et al., 2020; Queiroz Zetune Villa Real et al., 2023). Furthermore, co-expression of these intrabodies fused to distinct GEFTs allows for multi-color imaging of different synaptic proteins within the same neurons (Son et al., 2016). However, the intrabody-based approach has some caveats. Currently, specific intrabodies are available for only a limited number of synaptic proteins. Generating new intrabodies against a specific protein is costly, time-consuming, and labor-intensive, hindering the expansion of the library of intrabodies for various synaptic proteins. Even with available synaptic protein-targeting intrabodies, insufficient penetration into densely packed synaptic structures can impair accurate synaptic protein quantification. Furthermore, intrabody-based labeling of synaptic proteins can cause abnormal synaptic functions within a living neuron by disrupting the molecular conformation or interaction of these proteins (Marchionni et al., 2009). Therefore, individual intrabodies require careful validations before use.

### 3.2 Single-cell KI-mediated endogenous protein labeling

Endogenous protein tagging using GEFT knock-in (KI) is another powerful approach for quantitative mapping of endogenous proteins in brain tissue, as demonstrated in brain-wide synaptome analyses by Zhu et al. (2018), Cizeron et al. (2020), Bulovaite et al. (2022), Tomas-Roca et al. (2022), and Koukaroudi et al. (2024). Direct fusion of GEFTs to target proteins via genetic KI allows for spatially precise and quantitative measurements of fluorescent signals from GEFT-labeled proteins, with expression levels controlled by endogenous promoters. Although GEFT labeling of target proteins may potentially affect their native functions, previous studies have validated some tagging sites for each protein with minimal effects on its function. Artificial intelligence-based predictions of protein structures, such as the AlphaFold, may also be helpful (Jumper et al., 2021). Currently, two major approaches are available for single-cell GEFT KI in the brain.

#### 3.2.1 Conditional KI mouse

Cre-loxP-mediated conditional KI mice enable single-cell endogenous protein tagging with GEFTs in the brain. A duplicated exon strategy (called “ENABLED”) facilitates the generation of conditional KI mouse lines for GEFT tagging of target proteins at the C-terminus (Fortin et al., 2014). This method duplicates the stop-codon-containing exon into floxed/untagged and GEFT-tagged ones, allowing for a Cre-dependent transcription of the GEFT-tagged exon. Zhong and colleagues generated a conditional KI mouse line in which the C-terminus of PSD95 was tagged with GEFTs in a Cre-dependent manner (Fortin et al., 2014). A sparse introduction of the Cre gene into this mouse line provides single-cell labeling of PSD95 with GEFTs in both living and fixed brains.

A couple of technical limitations have disrupted applications of the ENABLED strategy to other proteins. First, the generation of conditional KI mouse lines is lower-throughput compared with that of knockout models. This productivity might be enhanced by the improved genome editing via oviductal nucleic acid delivery (i-GONAD) (Gurumurthy et al., 2016; Abe et al., 2023). Second, it may be difficult to apply the ENABLED-based KI design to regions of target proteins other than the C-terminus. Duplicated exons of target genes can be both spliced in at mRNA levels, consequently leading to an unnecessary amino acid insertion or frame-shift-mediated pre-mature termination at protein levels. Third, additional exons involved in the ENABLED strategy can cause abnormal transcription and translation of target genes in the absence of Cre recombinase. Indeed, PSD95-ENABLED mice exhibited the down-regulation of untagged PSD95 at both mRNA and protein levels (Fortin et al., 2014).

#### 3.2.2 *In vivo* genome editing-mediated KI

*In vivo* genome editing-mediated KI is also a promising approach for single-cell endogenous protein labeling. This technique leverages the cell-intrinsic DNA repair machinery in response to target-specific DNA double-strand breaks (DSBs), which are introduced by a Clustered Regularly Interspaced Short Palindromic Repeat (CRISPR)/CRISPR-associated protein 9 (Cas9) complex composed of a target-specific guide RNA (gRNA) and the Cas9 endonuclease protein (Jinek et al., 2012; Cong et al., 2013; Ran et al., 2013; Doudna and Charpentier, 2014). Two major DNA repair mechanisms, non-homologous end joint (NHEJ) and homology-directed repair (HDR), are frequently utilized for genome editing-mediated KI of GEFTs in neuronal tissues.

##### 3.2.2.1 NHEJ-mediated KI

NHEJ directly ligates pairs of free DNA ends at DSB sites (Lieber, 2010). Since the NHEJ machinery is active during both mitotic and non-mitotic phases of the cell cycle, it can repair CRISPR/Cas9-induced DNA DSBs with high efficiency in non-dividing neurons. Leveraging this DNA repair machinery, donor DNA fragments encoding tag sequences without any homology arms can be inserted at CRISPR/Cas9-induced DSB sites, enabling highly-efficient GEFT KI in neurons through NHEJ-mediated repair. This method is known as homology-independent targeted integration (HITI, Figure 1B) (Suzuki et al., 2016). The HITI technique is based on a DNA vector- or adeno-associated virus (AAV)-mediated delivery of target gene-specific gRNA and in-frame donor sequences into primary neuronal cultures, organotypic slices, or living brains. To enhance the generalizability of the HITI technique, a series of validated gRNA sequences have been demonstrated for mouse genes encoding 15 and 38 neuronal proteins as part of the HITI-based genome editing toolboxes, HiUGE and ORANGE, respectively (Table 1) (Gao et al., 2019; Willems et al., 2020). The HiUGE toolbox further offers a universal design for donor sequences that can be integrated into virtually any genomic target loci.

**Table 1:** List of proteins targeted by *in vivo* genome editing-mediated KI in the mouse.

Protein name	Category	Site	Mechanism	Reference
β-Actin	Cytoskeleton	N	NHEJ	Willems et al. (2020)
		N	NHEJ (CRISPIE)	Zhong et al. (2021)
		N	HDR	Mikuni et al. (2016)
				Tsunekawa et al. (2016)
				Uemura et al. (2016)
				Nishiyama et al. (2017)
				Meyerink et al. (2022)
Doublecortin X	Cytoskeleton	C	NHEJ	Gao et al. (2019)
		N	HDR	Mikuni et al. (2016)
GFAP	Cytoskeleton	C	NHEJ	Gao et al. (2019)
		C	NHEJ	Gao et al. (2019)
		N	NHEJ	Gao et al. (2019)
		N	HNEJ (CRISPIE)	Zhong et al. (2021)
		C	NHEJ	Suzuki et al. (2016)
				Tsunekawa et al. (2016)
				Willems et al. (2020)
				Gao et al. (2019)
		C	HDR (SATI)	Suzuki et al. (2019)
Vinculin	Cytoskeleton	C	NHEJ (CRISPIE)	Zhong et al. (2021)
Clathrin light chain	Endocytosis	N	NHEJ	Willems et al. (2020)
		C	NHEJ	Gao et al. (2019)
Rab11	Endocytosis	N	NHEJ	Willems et al. (2020)
		N	HDR	Mikuni et al. (2016)
14-3-3ε	Signaling	N	HDR	Mikuni et al. (2016)
Arhgap32	Signaling	C	NHEJ	Gao et al. (2019)
Arpc5	Signaling	C	NHEJ	Willems et al. (2020)
CaMKIIα	Signaling	N	NHEJ	Willems et al. (2020)
		C	NHEJ (CRISPIE)	Zhong et al. (2021)
		N/C	HDR	Mikuni et al. (2016)
				Nishiyama et al. (2017)
CaMKII*β*	Signaling	N/C	HDR	Mikuni et al. (2016)
CARMIL3	Signaling	C	NHEJ	Spence et al. (2019)
Erk2	Signaling	N	HDR	Nishiyama et al. (2017)
PKCα	Signaling	N	HDR	Mikuni et al. (2016)
WASP1	Signaling	C	NHEJ	Willems et al. (2020)
IgSF11	Adhesion	N	NHEJ	Hayano et al. (2021)
Nlgn3	Adhesion	N	NHEJ	Willems et al. (2020)
		N	NHEJ (TKIT)	Qin et al. (2024)
Nrcam	Adhesion	C	NHEJ	Gao et al. (2019)
CAPS1	Exocytosis	N	NHEJ	Willems et al. (2020)
Complexin1	Exocytosis	C	NHEJ	Willems et al. (2020)
Complexin2	Exocytosis	C	NHEJ	Willems et al. (2020)
Doc2a	Exocytosis	C	NHEJ	Willems et al. (2020)
Syt7	Exocytosis	N	NHEJ	Willems et al. (2020)
Bassoon	Active zone	N/C	NHEJ	Willems et al. (2020)
Munc13-1	Active zone	C	NHEJ	Willems et al. (2020)
Piccolo	Active zone	N	NHEJ	Willems et al. (2020)
RIM1	Active zone	C	NHEJ	Willems et al. (2020)
RIM2	Active zone	C	NHEJ	Willems et al. (2020)
Ankyrin-G	AIS	C	NHEJ	Gao et al. (2019)
βIV-Spectrin	AIS	C	NHEJ	Gao et al. (2019)
BK	Ion channel	C	NHEJ	Droogers et al. (2022)
Cav1.2	Ion channel	C	HDR	Mikuni et al. (2016)
Cav2.1 (P/Q)	Ion channel	N	NHEJ	Willems et al. (2020)
Cav2.3 (R)	Ion channel	N	NHEJ	Willems et al. (2020)
Cavβ1	Ion channel	N	NHEJ	Willems et al. (2020)
Cavβ2	Ion channel	C	NHEJ	Willems et al. (2020)
Cavβ3	Ion channel	C	NHEJ	Willems et al. (2020)
Cavβ4	Ion channel	C	NHEJ	Willems et al. (2020)
Scn2a	Ion channel	C	NHEJ	Gao et al. (2019)
SK2	Ion channel	C	NHEJ	Droogers et al. (2022)
Actr2	Postsynapse	C	NHEJ	Gao et al. (2019)
FRRS1L	Postsynapse	C	NHEJ	Willems et al. (2020)
Gephyrin	Postsynapse	N	NHEJ (TKIT)	Fang et al. (2021)
GSG1L	Postsynapse	C	NHEJ	Willems et al. (2020)
Homer1	Postsynapse	N	NHEJ (CRISPIE)	Droogers et al. (2022)
MPP2	Postsynapse	N	NHEJ	Droogers et al. (2022)
PSD95	Postsynapse	C	NHEJ	Willems et al. (2020)
Shank1	Postsynapse	C	NHEJ	Willems et al. (2020)
Shank2	Postsynapse	C	NHEJ	Willems et al. (2020)
TARPγ2	Postsynapse	C	NHEJ	Willems et al. (2020)
TARPγ8	Postsynapse	C	NHEJ	Willems et al. (2020)
GluA1	Receptor	N	NHEJ (TKIT)	Fang et al. (2021)
		C	NHEJ	Willems et al. (2020)
GluA2	Receptor	N	NHEJ (TKIT)	Fang et al. (2021)
		C	NHEJ	Willems et al. (2020)
GluA3	Receptor	N	NHEJ (TKIT)	Fang et al. (2021)
		C	NHEJ	Willems et al. (2020)
GluN1	Receptor	N	NHEJ (TKIT)	Fang et al. (2021)
		C	NHEJ	Willems et al. (2020)
GluN2A	Receptor	N	NHEJ (TKIT)	Fang et al. (2021)
		C	NHEJ	Willems et al. (2020)
GluN2B	Receptor	N	NHEJ (TKIT)	Fang et al. (2021)
		C	NHEJ	Willems et al. (2020)
GABA_A_Rβ2	Receptor	N	NHEJ (TKIT)	Fang et al. (2021)
Notch1	Receptor	C	HDR	Meyerink et al. (2022)
Emerin	Nucleus	N	HDR	Meyerink et al. (2022)
Laminin B1	Nucleus	N	HDR	Meyerink et al. (2022)
MeCP2	Nucleus	N/C	HDR	Mikuni et al. (2016)
		C	NHEJ	Gao et al. (2019)
Arc	Other	N	HDR	Mikuni et al. (2016)
FMRP	Other	N	HDR	Mikuni et al. (2016)
		C	HDR	Meyerink et al. (2022)
Pdha1	Other	C	NHEJ	Gao et al. (2019)
Rpl22	Other	C	HDR	Meyerink et al. (2022)

N and C indicates endogenous protein tagging at the N- and C-terminus, respectively. Some proteins described with N are tagged behind the signal sequence. Actr2, Actin related protein 2; AIS, axon initial segment; Arc, Activity-regulated cytoskeleton-associated protein; Arhgap32, Rho GTPase activating protein 32; Arpc5, Actin-related protein 2/3 complex subunit 5; BK, BK channel; CaMKIIα, Calcium/calmodulin-dependent protein kinase type II subunit alpha; CAPS1, calcium-dependent activator protein for secretion 1; CARMIL3, Capping protein regulator and myosin 1 linker 3; Cav, Voltage-dependent Ca2 + −channel; Doc2a, Double C2 domain alpha; Erk2; Extracellular signal-regulated kinase 2; FMRP, Fragile X messenger ribonucleotideprotein 1; FRRS1L, Ferric-chelate reductase 1-like protein; GFAP, Glial fibrillary acidic protein; GluA, Glutamate ionotropic receptor AMPA type subunit; GluN, Glutamate ionotropic receptor NMDA type subunit; GSG1L, Germ cell-specific gene 1-like protein; IgSF11, Immunoglobulin superfamily member 11; MAP2, Microtubule-associated protein 2; MPP2, Palmitoylated membrane protein 2; Nefm, Neurofilament medium; Nlgn3. Neuroligin-3; Nrcam, Neuronal cell adhesion molecule; Pdha1 pyruvate dehydrogenase E1 subunit alpha 1; PKCα, Protein kinase C alpha; PSD95, Postsynaptic protein 95; Rab11, Ras-related protein 11; RIM: Rab3-interacting molecule; Rpl22, Ribosomal Protein L22; Scn2a, Sodium voltage-gated channel alpha subunit 2; Shank, SH3 and multiple ankyrin repeat domains protein; SK2, SK2 channel; Syt7, synaptotagmin 7; TARP, Transmembrane AMPAR regulatory protein; WASP1, Wiskott-Aldrich syndrome protein 1.

HITI-based genome editing requires higher on-target and lower off-target activity of CRISPR/Cas9 complex. This can be achieved through optimized gRNA design (Doench et al., 2016; Xiang et al., 2021) and improved Cas9 derivatives, such as HiFi Cas9 and eSpCas9 (Slaymaker et al., 2016; Vakulskas et al., 2018). Since NHEJ is an error-prone DNA repair machinery, it can cause unfavorable DNA insertions or deletions (INDELs) at DSB sites (Lieber, 2010). Consequently, the HITI can cause INDELs within the coding sequence of the target genes, leading to nonsense or frame-shift mutations at protein levels. To mitigate this issue, the HITI has been modified to target CRISPR/Cas9-mediated DSBs in non-coding regions of the target genes. The CRISPR-mediated insertion of Exon (CRISPIE) inserts a designed exon-like donor, which consists of an exon encoding a tag sequence flanked by introns, into an intronic site in the target gene (Figure 1C) (Zhong et al., 2021). INDELs involved in the insertion of CRISPIE donors can be spliced out, leading to no INDELs at mRNA levels. However, an amino acid position to insert a tag sequence in the target protein depends on the exon-intron structure of the corresponding gene. Alternatively, Targeted Knock-In with Two guides (TKIT) makes CRISPR/Cas9-mediated DSBs at two intronic or non-coding sites, flanking a coding exon of the target gene to be replaced with a tagged one (Figure 1D) (Fang et al., 2021). This allows for an efficient, scarless tag KI at any target genomic loci. For instance, the TKIT provided a precise insertion of pH-sensitive fluorescent proteins a few amino acids downstream of the signal sequence of ionotropic glutamate receptors (Table 1). However, this strategy could cut off the target coding region flanked by two non-coding DSB sites or insert a designed exon at either DSB site (Danner et al., 2021), potentially resulting in a knockout of the target gene at the protein level. Another caveat of NHEJ-mediated genome editing is its poor compatibility with multiplex targeting of different genes in the same cells due to possible crosstalk between target gene-specific donors. Inducible Cre-mediated sequential targeting of different genes, which is named the Conditional Activation of KI Expression (CAKE), might solve this issue, while thorough optimizations of experimental conditions would be necessary for effective multiple targeting of distinct genes (Droogers et al., 2022).

##### 3.2.2.2 HDR-mediated KI

HDR is based on homologous recombination with donor DNA templates composed of two homology arms, allowing for a precise DNA repair (Filippo et al., 2008). However, since HDR is limited to the mitotic S and G2 phases of the cell cycle, it was thought to be inapplicable to non-dividing neurons. Mikuni et al. overcome this limitation by inducing CRISPR/Cas9-mediated HDR in neuronal progenitors which retain the ability of cell proliferations at the embryonic stage by *in utero* electroporation (IUE) (Figure 1E) (Mikuni et al., 2016). This method, termed SLENDR, allows for single-cell mEGFP KI for *CaMKIIα* and *CaMKIIβ* genes in the mouse neocortex. Likewise, other groups reported IUE-based HDR for tagging *β-Actin, βIII-Tubulin*, and *Laminin B1* genes with mEGFP or mCherry in cortical progenitors (Tsunekawa et al., 2016; Uemura et al., 2016; Kurihara et al., 2020; Meyerink et al., 2022). Interestingly, single homology arm donors can also efficiently induce error-free, HDR-like mEGFP KI for *βIII-Tubulin* gene, which is termed intercellular linearized Single homology Arm donor mediated intron-Targeting Integration (SATI) (Suzuki et al., 2019). The HDR-mediated KI efficiency for GEFTs was reported to range from 1–50%, which is highly variable depending on target genes, donor DNA templates, and the timing of IUE. This KI efficiency can be improved by manipulating the DNA repair machinery (Jayathilaka et al., 2008; Kurihara et al., 2020). Unlike NHEJ-mediated methods, CRISPR/Cas9-mediated HDR enables simultaneous targeting of multiple different genes, thereby increasing the generalizability of HDR-mediated endogenous protein labeling.

IUE-based introduction of CRISPR/Cas9-mediated HDR limits its accessibility to specific brain regions and embryonic stages. To address this limitation, Nishiyama et al. expanded CRISPR/Cas9-mediated HDR to post-mitotic neurons in various ages, cell types, and brain regions. This method is based on a high titer AAV-mediated transduction with gRNA and homology donor templates, which is called vSLENDR (Nishiyama et al., 2017). HDR-mediated genome editing can potentially lead to a leaky expression of a tag sequence from the donor template, possibly due to promoter activity in the 5′ homology arm. A polyadenylation sequence, which terminates the transcription, can suppress this leaky tag expression with its insertion upstream of the 5′ homology arm in a donor DNA template (Tsunekawa et al., 2016).

## 4 Discussion

Recent technological advances in genetic tools and genome editing methodologies have led to the development of various techniques for single-cell endogenous protein labeling in the mammalian brain. More than 70 protein species, including key postsynaptic scaffolds or receptors crucial for synaptic plasticity, have been validated in the mouse neuronal culture or brain tissue (Table 1). These resources not only facilitate mapping of the subcellular distribution of endogenous proteins in individual neurons, but also enable interrogation of the single-cell synaptome in the mouse brain.

Single-cell synaptome mapping is valuable for examining the synaptic and cellular mechanisms that underlie circuit remodeling during the critical period. This method can detect unique subpopulations of synapses, such as silent synapses, which are thought to serve as cellular substrates for circuit remodeling during the critical period (Huang et al., 2015). The spatiotemporal architecture of the single-cell synaptome may correlate with the activity or function of the same neuron, which can be captured by activity-dependent labeling with artificial promoters (ESARE, RAM) (Kawashima et al., 2013; Sorensen et al., 2016), immediate early genes (*c-fos*, *egr-1*, *Arc*) (Minatohara et al., 2016), or calcium indicators (CaMPARI2) (Moeyaert et al., 2018). This correlation would help us understand activity-dependent changes in synaptic diversity at the single-neuron level, which underlie circuit remodeling during the critical period. Furthermore, advanced GEFT technology enables single-cell mapping of not only protein localization but also protein dynamics in the brain. For instance, chemical tags that irreversibly bind to fluorescently-labeled ligands (Hoelzel and Zhang, 2020) allow for mapping of protein half-lives in the brain through monitoring the decrease in the pulse-label signals for pre-existing proteins fused to chemical tags (Bulovaite et al., 2022). Importantly, single-cell synaptome mapping is applicable to broad areas of neuroscience, such as neurodegenerative diseases and traumatic brain injuries, whose pathophysiological mechanisms involve the remodeling of individual synapses (Benarroch, 2018; Jamjoom et al., 2021).

In summary, single-cell endogenous protein labeling technologies, such as intrabodies, conditional KI, and genome-editing-mediated KI, provide a technical basis for molecular profiling of individual synapses within a single neuron in the mammalian brain tissue. This single-cell synaptome mapping would be a powerful and versatile approach for interrogating synaptic diversity within a single neuron in the brain, allowing for comprehensive and integrative understanding of the synaptic and cellular mechanisms that underpins circuit remodeling involved in neurodevelopmental and pathophysiological plasticity.
